# 6-(Hex-5-en­yloxy)naphthalene-2-carb­oxy­lic acid

**DOI:** 10.1107/S1600536814010642

**Published:** 2014-05-24

**Authors:** Md. Lutfor Rahman, H. T. Srinivasa, Mashitah Mohd. Yusoff, Huey Chong Kwong, Ching Kheng Quah

**Affiliations:** a University Malaysia Pahang, Faculty of Industrial Sciences and Technology, 26300 Gambang, Kuantan, Pahang, Malaysia; b Raman Research Institute, C.V. Raman Avenue, Sadashivanagar, Bangalore 560 080, India; c School of Chemical Sciences, Universiti Sains Malaysia, 11800 USM, Penang, Malaysia; d X-ray Crystallography Unit, School of Physics, Universiti Sains Malaysia, 11800 USM, Penang, Malaysia

## Abstract

The asymmetric unit of the title compound, C_17_H_18_O_3_, comprises three independent mol­ecules with similar geometries. In each mol­ecule, the carbonyl group is twisted away from the napthalene ring system, making dihedral angles of 1.0 (2), 1.05 (19)° and 1.5 (2)°. The butene group in all three mol­ecules are disordered over two sets of sites, with a refined occupancy ratio of 0.664 (6):0.336 (6). In the crystal, mol­ecules are oriented with respect to their carbonyl groups, forming head-to-head dimers *via* O—H⋯O hydrogen bonds. Adjacent dimers are further inter­connected by C—H⋯O hydrogen bonds into chains along the *a-*axis direction. The crystal structure is further stabilized by weak C—H⋯π inter­actions.

## Related literature   

For liquid crystal properties of carbonyl and naphthalene derivatives, see: Lee *et al.* (2001 ▶); Drzewinski (2013 ▶); Achalkumar *et al.* (2011 ▶). For naphthalene carb­oxy­lic acid derivatives, see: Rahman *et al.* (2013 ▶); Kozmik *et al.* (2005 ▶). For the synthesis of the title compound, see: Gopalakrishnan & Sadashiva (1998 ▶). For related structures, see: Fitzgerald & Gerkin (1993 ▶); Blackburn & Gerkin (1997 ▶); Lynch *et al.* (1998 ▶). For hydrogen-bond motifs, see: Bernstein *et al.* (1995 ▶). For bond-length data, see: Allen *et al.* (1987 ▶).
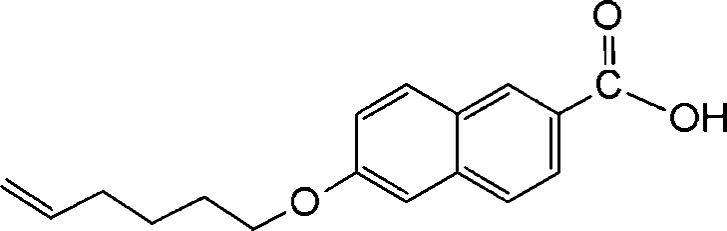


## Experimental   

### 

#### Crystal data  

C_17_H_18_O_3_
*M* *_r_* = 270.31
Triclinic, 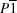

*a* = 9.5018 (2) Å
*b* = 14.8695 (2) Å
*c* = 17.6757 (3) Å
α = 113.638 (1)°
β = 102.188 (1)°
γ = 93.127 (1)°
*V* = 2209.06 (7) Å^3^
*Z* = 6
Cu *K*α radiation
μ = 0.67 mm^−1^
*T* = 298 K
0.53 × 0.21 × 0.18 mm

#### Data collection  

Bruker APEX DUO CCD area-detector diffractometer
Absorption correction: multi-scan (*SADABS*; Bruker, 2009 ▶) *T*_min_ = 0.719, *T*_max_ = 0.890
25536 measured reflections
6763 independent reflections
5768 reflections with *I* > 2σ(*I*)
*R*_int_ = 0.020

#### Refinement  

*R*[*F* ^2^ > 2σ(*F* ^2^)] = 0.045
*wR*(*F* ^2^) = 0.152
*S* = 1.06
6763 reflections
580 parameters
12 restraints
H-atom parameters constrained
Δρ_max_ = 0.23 e Å^−3^
Δρ_min_ = −0.17 e Å^−3^

### 

Data collection: *APEX2* (Bruker, 2009 ▶); cell refinement: *SAINT* (Bruker, 2009 ▶); data reduction: *SAINT*; program(s) used to solve structure: *SHELXTL* (Sheldrick, 2008 ▶); program(s) used to refine structure: *SHELXTL*; molecular graphics: *Mercury* (Macrae *et al.*, 2006 ▶); software used to prepare material for publication: *SHELXTL* and *PLATON* (Spek, 2009 ▶).

## Supplementary Material

Crystal structure: contains datablock(s) I. DOI: 10.1107/S1600536814010642/kp2470sup1.cif

Structure factors: contains datablock(s) a. DOI: 10.1107/S1600536814010642/kp2470Isup2.hkl

Supporting information file. DOI: 10.1107/S1600536814010642/kp2470Isup3.cml

CCDC references: 1001958, 1001959

Additional supporting information:  crystallographic information; 3D view; checkCIF report

## Figures and Tables

**Table 1:** Hydrogen-bond geometry (Å, °)

*D*—H⋯*A*	*D*—H	H⋯*A*	*D*⋯*A*	*D*—H⋯*A*
O2*A*—H1⋯O1*C* ^i^	0.97	1.65	2.6150 (16)	174
O2*B*—H2⋯O1*B* ^ii^	0.85	1.80	2.6342 (15)	168
O2*C*—H3⋯O1*A* ^i^	0.93	1.69	2.6133 (16)	177
C6*A*—H6*AA*⋯O1*A* ^iii^	0.93	2.50	3.3032 (19)	144
C6*B*—H6*BA*⋯O1*B* ^iii^	0.93	2.56	3.3666 (19)	145
C6*C*—H6*CA*⋯O1*C* ^iii^	0.93	2.56	3.3547 (18)	144
C5*A*—H5*AA*⋯O2*C* ^iv^	0.93	2.59	3.421 (2)	149
C5*B*—H5*BA*⋯O2*B* ^v^	0.93	2.65	3.520 (2)	156
C5*C*—H5*CA*⋯O2*A* ^iv^	0.93	2.61	3.472 (2)	154
C17*B*—H17*C*⋯*Cg*1^vi^	0.93	2.93	3.736 (5)	146

Symmetry codes: (i) 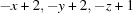; (ii) 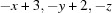; (iii) 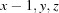; (iv) 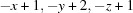; (v) 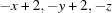; (vi) 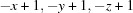.

**Fig. 1.**
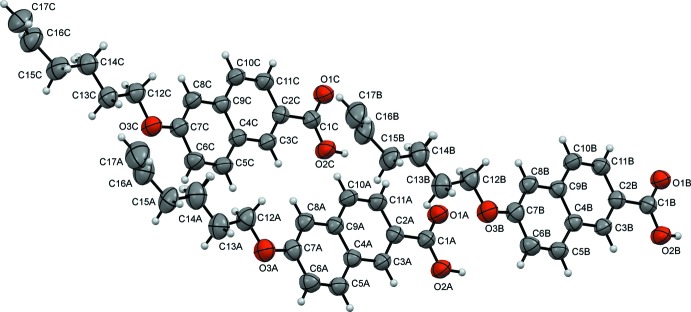
The molecular structure of the title compound, showing 30% probability displacement ellipsoids. Disordered butene groups (molecules A, B and C) with higher population components are shown.

**Fig. 2.**
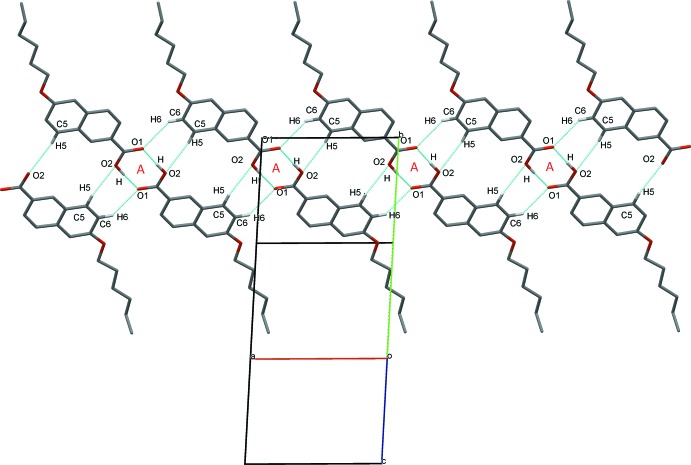
Part of the crystal packing of the title compound. Blue dashed lines represent the intermolecular hydrogen bonds and atoms involved in interactions are labelled. Disordered butene groups (in all molecules of asymmetric unit) with higher population components are shown. The symbol A represent the R22(8) graph-set motifs.

**Fig. 3.**
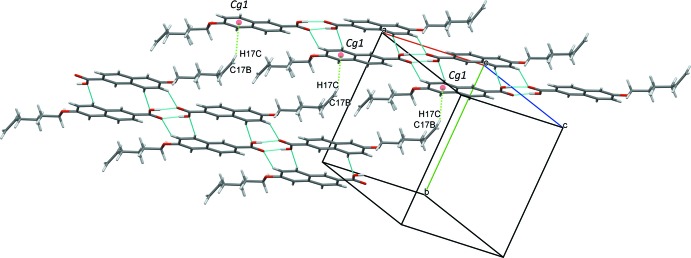
Part of the crystal packing of the title compound. Blue dashed lines represent the intermolecular hydrogen bonds within a layer and the green dashed lines represent the weak intermolecular C17B—H17C···Cg1 interaction (Cg1 is the centroid of the C4C—C9C).

## supplementary crystallographic information

### 1. Comment

The title compound is considered a potential candidate for material chemistry study comprising a polymerizable vinyl group at one end and a free carboxylic acid group at the other end of the molecule. In general, the free carboxylic acid group favours to form the hydrogen-bonded cyclic dimers in the liquid crystalline phases and most of the dimers exhibited enantiotropic liquid crystalline behavior (Lee *et al.* 2001). Considerable amount of work has been carried out in naphthalene derivatives to achieve application oriented SmC phase in low molar mass and polymeric liquid crystals with an azo/ester group (Drzewinski 2013; Achalkumar *et al.* 2011). These materials often studied in the view of their interesting optical properties, which enable applications such as optical switching, holography and optical storage devices (Rahman *et al.* 2013; Kozmik *et al.* 2005). This paper presents synthesis and crystal structure analysis of naphthalene based liquid crystalline precursor.

The asymmetry unit of the title compound (Fig. 1) comprises three crystallographically independent molecules (*A*, *B* and *C*) of similar geometries. The bonds lengths (Allen *et al.* 1987) and angles have normal values and comparable with the closely related structures (Fitzgerald & Gerkin 1993; Blackburn & Gerkin 1997; Lynch *et al.* 1998). In each molecule, the carbonyl group is almost coplanar with the attached naphthalene ring. The carbonyl group (O1—C1—O2) is slightly twisted away from the naphthalene ring system, with the dihedral angles of 1.00 (20)° in molecule *A*, 1.05 (19)° in molecule *B* and 1.50 (20)° in molecule *C*]. The butene groups which attached to atom C14 in each molecule (*A*, *B* and *C*) are disordered over two positions with a refined site-occupancy ratio of 0.695 (6): 0.305 (6).

In the crystal packing (Fig. 2), two adjacent molecules are linked into inversion dimers, forming *R*^2^_2_(8) graph-set motifs (Bernstein *et al.* 1995) by a pair of intermolecular O2—H···O1 hydrogen bond (Table 1). These dimers are linked into chain along the *a* axis *via* intermolecular C5—H5···O2 and C6—H6···O1 hydrogen bonds. The crystal structure is further stabilized by a weak intermolecular C17B—H17C···*Cg*1 interaction (*Cg*1 is the centroid of the C4C—C9,*C* Table 1).

### 2. Experimental

The title compound is synthesized according to the literature (Gopalakrishnan & Sadashiva 1998). Pure and suitable single crystals were obtained on slow evaporation of ethyl alcohol at room temperature. The compound melts at 419 K to nematic phase, then it goes to isotropic state at 461 K. It returns to nematic phase at 486 K and then crystallizes at 410 K when it was cooled from isotropic state.

### 3. Refinement

The butene group and hydrogen atoms which are attached to atom C14 in each molecule (*A*, *B* and *C*) are disordered over two positions with a refined site-occupancy ratio of 0.695 (6): 0.305 (6). All C-bound H atoms were positioned geometrically [C—H = 0.95–0.97 Å] and refined using a riding model with *U*_iso_(H) = 1.2 or 1.5 *U*_eq_(C). All O-bound H atoms were located from difference Fourier map and were fixed to their parent atoms with *U*_iso_(H) = 1.5 *U*_eq_(O). The restraints of same geometries were applied to all disordered components. Three outliners, (1 - 3 2), (-3 2 0) and (0 3 3), were omitted.

### Crystal data

**Table:**

C_17_H_18_O_3_	*Z* = 6
*M**_r_* = 270.31	*F*(000) = 864
Triclinic, *P*1	*D*_x_ = 1.219 Mg m^−^^3^
Hall symbol: -P 1	Cu *K*α radiation, λ = 1.54178 Å
*a* = 9.5018 (2) Å	Cell parameters from 9936 reflections
*b* = 14.8695 (2) Å	θ = 2.8–69.4°
*c* = 17.6757 (3) Å	µ = 0.67 mm^−^^1^
α = 113.638 (1)°	*T* = 298 K
β = 102.188 (1)°	Block, colourless
γ = 93.127 (1)°	0.53 × 0.21 × 0.18 mm
*V* = 2209.06 (7) Å^3^	

### Data collection

**Table:**

Bruker APEX DUO CCD area-detector diffractometer	6763 independent reflections
Radiation source: fine-focus sealed tube	5768 reflections with *I* > 2σ(*I*)
Graphite monochromator	*R*_int_ = 0.020
φ and ω scans	θ_max_ = 62.5°, θ_min_ = 2.8°
Absorption correction: multi-scan (*SADABS*; Bruker, 2009)	*h* = −10→10
*T*_min_ = 0.719, *T*_max_ = 0.890	*k* = −17→17
25536 measured reflections	*l* = −20→17

### Refinement

**Table:**

Refinement on *F*^2^	Primary atom site location: structure-invariant direct methods
Least-squares matrix: full	Secondary atom site location: difference Fourier map
*R*[*F*^2^ > 2σ(*F*^2^)] = 0.045	Hydrogen site location: inferred from neighbouring sites
*wR*(*F*^2^) = 0.152	H-atom parameters constrained
*S* = 1.06	*w* = 1/[σ^2^(*F*_o_^2^) + (0.0897*P*)^2^ + 0.1917*P*] where *P* = (*F*_o_^2^ + 2*F*_c_^2^)/3
6763 reflections	(Δ/σ)_max_ = 0.001
580 parameters	Δρ_max_ = 0.23 e Å^−^^3^
12 restraints	Δρ_min_ = −0.17 e Å^−^^3^

### Special details

**Table:**

Geometry. All esds (except the esd in the dihedral angle between two l.s. planes) are estimated using the full covariance matrix. The cell esds are taken into account individually in the estimation of esds in distances, angles and torsion angles; correlations between esds in cell parameters are only used when they are defined by crystal symmetry. An approximate (isotropic) treatment of cell esds is used for estimating esds involving l.s. planes.
Refinement. Refinement of *F*^2^ against ALL reflections. The weighted *R*-factor *wR* and goodness of fit *S* are based on *F*^2^, conventional *R*-factors *R* are based on *F*, with *F* set to zero for negative *F*^2^. The threshold expression of *F*^2^ > σ(*F*^2^) is used only for calculating *R*-factors(gt) *etc*. and is not relevant to the choice of reflections for refinement. *R*-factors based on *F*^2^ are statistically about twice as large as those based on *F*, and *R*- factors based on ALL data will be even larger.

### Fractional atomic coordinates and isotropic or equivalent isotropic displacement parameters (Å^2^)

**Table:**

	*x*	*y*	*z*	*U*_iso_*/*U*_eq_	Occ. (<1)
C11C	0.95456 (14)	0.84092 (10)	0.81731 (8)	0.0590 (3)	
H11A	1.0541	0.8394	0.8316	0.071*	
O1A	0.88492 (10)	0.95388 (8)	0.38257 (7)	0.0761 (3)	
O2A	0.69392 (11)	0.99235 (8)	0.31169 (7)	0.0755 (3)	
H1	0.7582	1.0190	0.2870	0.113*	
C2B	1.27582 (13)	0.89459 (9)	0.07820 (8)	0.0514 (3)	
C11B	1.33200 (14)	0.84821 (10)	0.13154 (8)	0.0575 (3)	
H11B	1.4315	0.8462	0.1453	0.069*	
O1B	1.50686 (10)	0.93180 (7)	0.05874 (7)	0.0686 (3)	
O2B	1.31640 (10)	0.98007 (7)	−0.00396 (7)	0.0720 (3)	
H2	1.3830	1.0058	−0.0179	0.108*	
C9B	1.08934 (13)	0.80896 (9)	0.14448 (8)	0.0511 (3)	
O1C	1.13044 (10)	0.92441 (8)	0.74563 (7)	0.0702 (3)	
C2A	0.65249 (14)	0.91504 (9)	0.39971 (8)	0.0533 (3)	
C9C	0.71133 (13)	0.80165 (9)	0.82925 (8)	0.0526 (3)	
O2C	0.94033 (10)	0.96819 (8)	0.67871 (7)	0.0754 (3)	
H3	1.0014	0.9978	0.6580	0.113*	
C4B	1.03382 (13)	0.85815 (9)	0.09276 (8)	0.0512 (3)	
C7C	0.46924 (14)	0.76419 (10)	0.84158 (9)	0.0585 (3)	
C2C	0.89811 (13)	0.88579 (9)	0.76267 (8)	0.0523 (3)	
C14A	0.0353 (2)	0.62196 (14)	0.62674 (12)	0.0919 (5)	
H14A	0.0887	0.5681	0.6030	0.110*	0.664 (6)
H14B	0.0969	0.6683	0.6817	0.110*	0.664 (6)
H14G	0.0882	0.6670	0.6844	0.110*	0.336 (6)
H14H	0.0870	0.5663	0.6055	0.110*	0.336 (6)
C4A	0.40916 (14)	0.87883 (9)	0.41207 (8)	0.0527 (3)	
C11A	0.70786 (14)	0.87345 (10)	0.45694 (8)	0.0599 (3)	
H11C	0.8074	0.8723	0.4721	0.072*	
C7B	0.84757 (15)	0.77311 (10)	0.15801 (9)	0.0596 (3)	
C4C	0.65552 (13)	0.84871 (9)	0.77598 (8)	0.0520 (3)	
C7A	0.22020 (15)	0.80107 (10)	0.47998 (9)	0.0625 (3)	
C6B	0.79310 (15)	0.82404 (10)	0.10851 (9)	0.0644 (4)	
H6BA	0.6946	0.8296	0.0976	0.077*	
C6C	0.41426 (14)	0.81381 (10)	0.79116 (9)	0.0643 (3)	
H6CA	0.3156	0.8188	0.7799	0.077*	
O3B	0.74330 (11)	0.73435 (8)	0.18358 (7)	0.0750 (3)	
C6A	0.16638 (15)	0.84526 (11)	0.42473 (9)	0.0664 (4)	
H6AA	0.0674	0.8487	0.4109	0.080*	
C3C	0.75144 (14)	0.88935 (9)	0.74318 (8)	0.0537 (3)	
H3CA	0.7148	0.9193	0.7075	0.064*	
O3C	0.36603 (10)	0.72389 (8)	0.86688 (7)	0.0695 (3)	
C3B	1.12942 (14)	0.89931 (9)	0.06024 (8)	0.0526 (3)	
H3BA	1.0926	0.9305	0.0257	0.063*	
C13C	0.27997 (15)	0.62535 (11)	0.93018 (9)	0.0655 (4)	
H13A	0.2178	0.5782	0.8757	0.079*	
H13B	0.2252	0.6776	0.9557	0.079*	
C13B	0.64881 (17)	0.64431 (12)	0.25026 (10)	0.0746 (4)	
H13C	0.5856	0.5976	0.1960	0.090*	
H13D	0.5976	0.6994	0.2744	0.090*	
C3A	0.50620 (14)	0.91728 (9)	0.37867 (8)	0.0537 (3)	
H3AA	0.4700	0.9450	0.3412	0.064*	
C10B	1.24113 (14)	0.80637 (10)	0.16294 (8)	0.0580 (3)	
H10A	1.2798	0.7754	0.1973	0.070*	
O3A	0.11378 (11)	0.76599 (8)	0.50651 (7)	0.0789 (3)	
C10C	0.86334 (14)	0.79986 (10)	0.84898 (8)	0.0598 (3)	
H10B	0.9018	0.7700	0.8843	0.072*	
C10A	0.61622 (15)	0.83494 (10)	0.49016 (9)	0.0610 (3)	
H10C	0.6544	0.8077	0.5276	0.073*	
C5B	0.88300 (14)	0.86486 (10)	0.07668 (9)	0.0603 (3)	
H5BA	0.8453	0.8978	0.0438	0.072*	
C5C	0.50437 (14)	0.85437 (10)	0.75895 (9)	0.0616 (3)	
H5CA	0.4664	0.8863	0.7253	0.074*	
C13A	0.01091 (18)	0.67434 (12)	0.56904 (11)	0.0807 (4)	
H13E	−0.0417	0.7289	0.5923	0.097*	
H13F	−0.0493	0.6284	0.5134	0.097*	
C1A	0.75063 (14)	0.95599 (9)	0.36308 (8)	0.0573 (3)	
C1C	0.99664 (14)	0.92866 (9)	0.72752 (8)	0.0560 (3)	
C8B	0.99318 (15)	0.76624 (10)	0.17631 (8)	0.0575 (3)	
H8BA	1.0286	0.7334	0.2097	0.069*	
C5A	0.25799 (14)	0.88260 (10)	0.39172 (9)	0.0620 (3)	
H5AA	0.2211	0.9112	0.3551	0.074*	
C1B	1.37349 (14)	0.93781 (9)	0.04264 (8)	0.0550 (3)	
C8C	0.61515 (14)	0.75945 (10)	0.86145 (8)	0.0577 (3)	
H8CA	0.6510	0.7283	0.8962	0.069*	
C9A	0.46405 (14)	0.83562 (9)	0.46872 (8)	0.0535 (3)	
C12C	0.41192 (15)	0.66930 (11)	0.91581 (10)	0.0650 (4)	
H12A	0.4781	0.7130	0.9702	0.078*	
H12B	0.4626	0.6169	0.8854	0.078*	
C12A	0.15113 (17)	0.71425 (12)	0.55827 (10)	0.0759 (4)	
H12C	0.2142	0.7588	0.6134	0.091*	
H12D	0.2019	0.6602	0.5311	0.091*	
C12B	0.78511 (16)	0.68236 (11)	0.23525 (10)	0.0698 (4)	
H12E	0.8519	0.7266	0.2892	0.084*	
H12F	0.8333	0.6275	0.2064	0.084*	
C8A	0.36617 (15)	0.79645 (10)	0.50215 (9)	0.0610 (3)	
H8AA	0.4006	0.7677	0.5390	0.073*	
C14C	0.32087 (17)	0.57320 (12)	0.98788 (11)	0.0754 (4)	
H14E	0.3880	0.6194	1.0409	0.090*	0.664 (6)
H14F	0.3706	0.5185	0.9606	0.090*	0.664 (6)
H14K	0.3715	0.6223	1.0446	0.090*	0.336 (6)
H14L	0.3877	0.5281	0.9665	0.090*	0.336 (6)
C14B	0.67968 (19)	0.59356 (13)	0.30987 (11)	0.0828 (5)	
H14C	0.7294	0.5380	0.2847	0.099*	0.664 (6)
H14D	0.7460	0.6401	0.3631	0.099*	0.664 (6)
H14I	0.7411	0.6387	0.3653	0.099*	0.336 (6)
H14J	0.7261	0.5357	0.2862	0.099*	0.336 (6)
C15A	−0.0974 (6)	0.5802 (3)	0.6415 (3)	0.0832 (11)	0.664 (6)
H15A	−0.1574	0.6319	0.6583	0.100*	0.664 (6)
H15B	−0.1530	0.5274	0.5878	0.100*	0.664 (6)
C16A	−0.0708 (5)	0.5402 (2)	0.7067 (3)	0.0916 (10)	0.664 (6)
H16A	−0.0137	0.5813	0.7610	0.110*	0.664 (6)
C17A	−0.1241 (6)	0.4492 (3)	0.6915 (3)	0.1266 (16)	0.664 (6)
H17A	−0.1816	0.4066	0.6376	0.152*	0.664 (6)
H17B	−0.1042	0.4275	0.7346	0.152*	0.664 (6)
C15B	0.5494 (6)	0.5560 (4)	0.3299 (3)	0.0813 (11)	0.664 (6)
H15C	0.4917	0.6092	0.3460	0.098*	0.664 (6)
H15D	0.4909	0.5025	0.2776	0.098*	0.664 (6)
C16B	0.5729 (5)	0.5199 (3)	0.3958 (3)	0.0875 (11)	0.664 (6)
H16B	0.6295	0.5635	0.4496	0.105*	0.664 (6)
C17B	0.5219 (6)	0.4323 (3)	0.3865 (3)	0.1046 (13)	0.664 (6)
H17C	0.4648	0.3863	0.3338	0.126*	0.664 (6)
H17D	0.5426	0.4154	0.4325	0.126*	0.664 (6)
C15C	0.1867 (6)	0.5328 (5)	1.0080 (4)	0.0738 (19)	0.664 (6)
H15E	0.1222	0.5823	1.0194	0.089*	0.664 (6)
H15F	0.1341	0.4739	0.9581	0.089*	0.664 (6)
C16C	0.2253 (3)	0.5076 (3)	1.0819 (3)	0.0928 (11)	0.664 (6)
H16C	0.2837	0.5577	1.1321	0.111*	0.664 (6)
C17C	0.1892 (4)	0.4272 (3)	1.0857 (3)	0.1042 (12)	0.664 (6)
H17E	0.1308	0.3743	1.0377	0.125*	0.664 (6)
H17F	0.2208	0.4207	1.1365	0.125*	0.664 (6)
C15X	−0.1273 (16)	0.5851 (10)	0.6242 (9)	0.114 (4)*	0.336 (6)
H15G	−0.1746	0.6422	0.6491	0.137*	0.336 (6)
H15H	−0.1822	0.5467	0.5654	0.137*	0.336 (6)
C16X	−0.1261 (15)	0.5260 (9)	0.6701 (8)	0.154 (5)*	0.336 (6)
H16D	−0.2039	0.5362	0.6953	0.185*	0.336 (6)
C17X	−0.0641 (15)	0.4698 (11)	0.6878 (9)	0.164 (5)*	0.336 (6)
H17G	0.0176	0.4509	0.6679	0.196*	0.336 (6)
H17H	−0.0957	0.4434	0.7220	0.196*	0.336 (6)
C15Y	0.5194 (14)	0.5626 (10)	0.3175 (9)	0.096 (4)*	0.336 (6)
H15I	0.4718	0.6205	0.3384	0.116*	0.336 (6)
H15J	0.4590	0.5154	0.2625	0.116*	0.336 (6)
C16Y	0.544 (2)	0.5186 (13)	0.3768 (11)	0.210 (10)*	0.336 (6)
H16E	0.5448	0.5665	0.4307	0.252*	0.336 (6)
C17Y	0.563 (2)	0.4435 (13)	0.3816 (13)	0.203 (9)*	0.336 (6)
H17I	0.5645	0.3875	0.3331	0.244*	0.336 (6)
H17J	0.5755	0.4401	0.4340	0.244*	0.336 (6)
C15Z	0.1984 (19)	0.5311 (13)	1.0046 (12)	0.112 (7)*	0.336 (6)
H15K	0.1227	0.4989	0.9516	0.135*	0.336 (6)
H15L	0.1603	0.5844	1.0440	0.135*	0.336 (6)
C16Z	0.2317 (7)	0.4548 (5)	1.0422 (4)	0.0839 (19)*	0.336 (6)
H16F	0.2543	0.3923	1.0122	0.101*	0.336 (6)
C17Z	0.2239 (9)	0.4900 (8)	1.1239 (5)	0.121 (3)*	0.336 (6)
H17K	0.2007	0.5533	1.1503	0.145*	0.336 (6)
H17L	0.2416	0.4511	1.1540	0.145*	0.336 (6)

### Atomic displacement parameters (Å^2^)

**Table:**

	*U*^11^	*U*^22^	*U*^33^	*U*^12^	*U*^13^	*U*^23^
C11C	0.0464 (7)	0.0740 (8)	0.0662 (8)	0.0126 (6)	0.0140 (6)	0.0389 (6)
O1A	0.0526 (6)	0.1063 (8)	0.1005 (8)	0.0207 (5)	0.0269 (5)	0.0701 (6)
O2A	0.0641 (6)	0.1051 (7)	0.0933 (7)	0.0256 (5)	0.0294 (5)	0.0716 (6)
C2B	0.0504 (7)	0.0533 (7)	0.0531 (7)	0.0082 (5)	0.0148 (5)	0.0243 (5)
C11B	0.0450 (7)	0.0713 (8)	0.0639 (8)	0.0115 (6)	0.0135 (6)	0.0360 (6)
O1B	0.0514 (6)	0.0867 (7)	0.0895 (7)	0.0153 (5)	0.0243 (5)	0.0552 (5)
O2B	0.0612 (6)	0.0923 (7)	0.0952 (7)	0.0218 (5)	0.0299 (5)	0.0662 (6)
C9B	0.0483 (7)	0.0538 (6)	0.0512 (7)	0.0060 (5)	0.0110 (5)	0.0233 (5)
O1C	0.0527 (6)	0.0904 (7)	0.0918 (7)	0.0181 (5)	0.0251 (5)	0.0586 (6)
C2A	0.0515 (7)	0.0572 (7)	0.0561 (7)	0.0110 (5)	0.0159 (6)	0.0274 (6)
C9C	0.0489 (7)	0.0572 (7)	0.0532 (7)	0.0076 (5)	0.0100 (5)	0.0265 (5)
O2C	0.0613 (6)	0.1042 (7)	0.0961 (7)	0.0216 (5)	0.0281 (5)	0.0726 (6)
C4B	0.0491 (7)	0.0527 (6)	0.0519 (7)	0.0066 (5)	0.0112 (5)	0.0232 (5)
C7C	0.0497 (7)	0.0647 (7)	0.0644 (8)	0.0047 (6)	0.0140 (6)	0.0315 (6)
C2C	0.0522 (7)	0.0551 (7)	0.0543 (7)	0.0098 (5)	0.0165 (5)	0.0263 (5)
C14A	0.0899 (12)	0.0975 (12)	0.0974 (12)	−0.0029 (9)	0.0364 (10)	0.0463 (10)
C4A	0.0503 (7)	0.0539 (7)	0.0543 (7)	0.0084 (5)	0.0127 (5)	0.0235 (5)
C11A	0.0485 (7)	0.0746 (8)	0.0672 (8)	0.0139 (6)	0.0149 (6)	0.0400 (6)
C7B	0.0500 (7)	0.0644 (7)	0.0662 (8)	0.0015 (6)	0.0156 (6)	0.0298 (6)
C4C	0.0492 (7)	0.0549 (7)	0.0543 (7)	0.0074 (5)	0.0114 (5)	0.0265 (5)
C7A	0.0559 (7)	0.0644 (8)	0.0701 (8)	0.0028 (6)	0.0219 (6)	0.0292 (6)
C6B	0.0464 (7)	0.0758 (9)	0.0763 (9)	0.0090 (6)	0.0140 (6)	0.0382 (7)
C6C	0.0464 (7)	0.0784 (9)	0.0780 (9)	0.0105 (6)	0.0139 (6)	0.0439 (7)
O3B	0.0539 (5)	0.0939 (7)	0.0946 (7)	0.0026 (5)	0.0226 (5)	0.0564 (6)
C6A	0.0494 (7)	0.0750 (9)	0.0782 (9)	0.0097 (6)	0.0144 (6)	0.0367 (7)
C3C	0.0550 (7)	0.0572 (7)	0.0552 (7)	0.0113 (5)	0.0125 (6)	0.0304 (6)
O3C	0.0497 (5)	0.0889 (7)	0.0882 (7)	0.0051 (4)	0.0189 (5)	0.0557 (5)
C3B	0.0533 (7)	0.0545 (7)	0.0550 (7)	0.0097 (5)	0.0132 (5)	0.0284 (5)
C13C	0.0582 (8)	0.0710 (8)	0.0728 (9)	0.0030 (6)	0.0192 (6)	0.0354 (7)
C13B	0.0701 (9)	0.0762 (9)	0.0803 (10)	−0.0026 (7)	0.0272 (8)	0.0335 (8)
C3A	0.0553 (7)	0.0581 (7)	0.0545 (7)	0.0113 (5)	0.0135 (5)	0.0307 (5)
C10B	0.0527 (7)	0.0715 (8)	0.0612 (7)	0.0126 (6)	0.0125 (6)	0.0400 (6)
O3A	0.0627 (6)	0.0947 (7)	0.0978 (8)	0.0065 (5)	0.0291 (5)	0.0555 (6)
C10C	0.0522 (7)	0.0753 (8)	0.0656 (8)	0.0127 (6)	0.0133 (6)	0.0440 (6)
C10A	0.0574 (7)	0.0743 (8)	0.0644 (8)	0.0156 (6)	0.0137 (6)	0.0429 (6)
C5B	0.0506 (7)	0.0696 (8)	0.0682 (8)	0.0118 (6)	0.0120 (6)	0.0379 (6)
C5C	0.0519 (7)	0.0726 (8)	0.0710 (8)	0.0132 (6)	0.0107 (6)	0.0429 (7)
C13A	0.0756 (10)	0.0819 (10)	0.0853 (10)	−0.0057 (8)	0.0282 (8)	0.0344 (8)
C1A	0.0553 (7)	0.0648 (7)	0.0633 (8)	0.0150 (6)	0.0196 (6)	0.0356 (6)
C1C	0.0548 (7)	0.0600 (7)	0.0594 (7)	0.0128 (6)	0.0174 (6)	0.0297 (6)
C8B	0.0549 (7)	0.0636 (7)	0.0611 (7)	0.0059 (6)	0.0138 (6)	0.0343 (6)
C5A	0.0535 (7)	0.0709 (8)	0.0693 (8)	0.0118 (6)	0.0132 (6)	0.0383 (6)
C1B	0.0533 (7)	0.0573 (7)	0.0612 (7)	0.0114 (6)	0.0182 (6)	0.0297 (6)
C8C	0.0525 (7)	0.0674 (8)	0.0634 (8)	0.0079 (6)	0.0126 (6)	0.0390 (6)
C9A	0.0537 (7)	0.0551 (7)	0.0553 (7)	0.0083 (5)	0.0146 (5)	0.0265 (5)
C12C	0.0577 (8)	0.0719 (8)	0.0743 (9)	0.0054 (6)	0.0172 (6)	0.0401 (7)
C12A	0.0738 (9)	0.0840 (10)	0.0780 (9)	0.0006 (8)	0.0248 (8)	0.0412 (8)
C12B	0.0651 (8)	0.0748 (9)	0.0764 (9)	0.0007 (7)	0.0213 (7)	0.0384 (7)
C8A	0.0620 (8)	0.0654 (8)	0.0634 (8)	0.0083 (6)	0.0166 (6)	0.0350 (6)
C14C	0.0631 (8)	0.0874 (10)	0.0939 (11)	0.0102 (7)	0.0261 (8)	0.0536 (9)
C14B	0.0829 (11)	0.0878 (11)	0.0914 (11)	0.0055 (8)	0.0365 (9)	0.0454 (9)
C15A	0.084 (2)	0.090 (2)	0.089 (2)	−0.0152 (17)	0.031 (2)	0.0489 (18)
C16A	0.095 (2)	0.098 (2)	0.092 (2)	−0.0039 (17)	0.032 (2)	0.0482 (17)
C17A	0.160 (4)	0.110 (3)	0.161 (4)	0.027 (3)	0.088 (3)	0.085 (2)
C15B	0.074 (2)	0.104 (2)	0.091 (3)	0.0020 (19)	0.030 (2)	0.063 (2)
C16B	0.089 (2)	0.105 (3)	0.0872 (19)	−0.0023 (15)	0.0335 (16)	0.0553 (16)
C17B	0.127 (3)	0.106 (2)	0.127 (3)	0.0277 (19)	0.069 (2)	0.076 (2)
C15C	0.0571 (18)	0.094 (3)	0.096 (3)	0.0009 (11)	0.0241 (15)	0.065 (2)
C16C	0.0880 (18)	0.121 (3)	0.098 (2)	0.0082 (16)	0.0299 (16)	0.072 (2)
C17C	0.122 (3)	0.110 (3)	0.118 (3)	0.028 (2)	0.042 (2)	0.079 (2)

### Geometric parameters (Å, º)

**Table:**

C11C—C10C	1.3634 (18)	C10C—H10B	0.9300
C11C—C2C	1.4151 (18)	C10A—C9A	1.4169 (19)
C11C—H11A	0.9300	C10A—H10C	0.9300
O1A—C1A	1.2551 (16)	C5B—H5BA	0.9300
O2A—C1A	1.2755 (15)	C5C—H5CA	0.9300
O2A—H1	0.9728	C13A—C12A	1.508 (2)
C2B—C3B	1.3722 (18)	C13A—H13E	0.9700
C2B—C11B	1.4138 (18)	C13A—H13F	0.9700
C2B—C1B	1.4746 (17)	C8B—H8BA	0.9300
C11B—C10B	1.3643 (18)	C5A—H5AA	0.9300
C11B—H11B	0.9300	C8C—H8CA	0.9300
O1B—C1B	1.2551 (16)	C9A—C8A	1.4149 (19)
O2B—C1B	1.2773 (15)	C12C—H12A	0.9700
O2B—H2	0.8501	C12C—H12B	0.9700
C9B—C8B	1.4125 (19)	C12A—H12C	0.9700
C9B—C10B	1.4162 (18)	C12A—H12D	0.9700
C9B—C4B	1.4224 (18)	C12B—H12E	0.9700
O1C—C1C	1.2554 (16)	C12B—H12F	0.9700
C2A—C3A	1.3669 (18)	C8A—H8AA	0.9300
C2A—C11A	1.4124 (18)	C14C—C15Z	1.430 (16)
C2A—C1A	1.4726 (18)	C14C—C15C	1.546 (5)
C9C—C8C	1.4129 (18)	C14C—H14E	0.9700
C9C—C10C	1.4169 (18)	C14C—H14F	0.9700
C9C—C4C	1.4197 (17)	C14C—H14K	0.9699
O2C—C1C	1.2736 (15)	C14C—H14L	0.9700
O2C—H3	0.9243	C14B—C15B	1.496 (4)
C4B—C3B	1.4043 (18)	C14B—C15Y	1.616 (14)
C4B—C5B	1.4180 (18)	C14B—H14C	0.9700
C7C—O3C	1.3628 (16)	C14B—H14D	0.9700
C7C—C8C	1.3695 (19)	C14B—H14I	0.9700
C7C—C6C	1.4085 (19)	C14B—H14J	0.9700
C2C—C3C	1.3724 (18)	C15A—C16A	1.478 (6)
C2C—C1C	1.4721 (18)	C15A—H15A	0.9700
C14A—C15A	1.493 (4)	C15A—H15B	0.9700
C14A—C13A	1.502 (2)	C16A—C17A	1.318 (6)
C14A—C15X	1.596 (14)	C16A—H16A	0.9300
C14A—H14A	0.9700	C17A—H17A	0.9300
C14A—H14B	0.9700	C17A—H17B	0.9300
C14A—H14G	0.9700	C15B—C16B	1.448 (5)
C14A—H14H	0.9700	C15B—H15C	0.9700
C4A—C3A	1.4032 (18)	C15B—H15D	0.9700
C4A—C5A	1.4151 (18)	C16B—C17B	1.299 (5)
C4A—C9A	1.4211 (18)	C16B—H16B	0.9300
C11A—C10A	1.3645 (19)	C17B—H17C	0.9300
C11A—H11C	0.9300	C17B—H17D	0.9300
C7B—O3B	1.3614 (17)	C15C—C16C	1.480 (5)
C7B—C8B	1.372 (2)	C15C—H15E	0.9700
C7B—C6B	1.411 (2)	C15C—H15F	0.9700
C4C—C3C	1.4056 (18)	C16C—C17C	1.257 (5)
C4C—C5C	1.4182 (18)	C16C—H16C	0.9300
C7A—O3A	1.3615 (17)	C17C—H17E	0.9300
C7A—C8A	1.373 (2)	C17C—H17F	0.9300
C7A—C6A	1.410 (2)	C15X—C16X	1.414 (12)
C6B—C5B	1.355 (2)	C15X—H15G	0.9700
C6B—H6BA	0.9300	C15X—H15H	0.9700
C6C—C5C	1.3585 (19)	C16X—C17X	1.152 (13)
C6C—H6CA	0.9300	C16X—H16D	0.9300
O3B—C12B	1.4269 (18)	C17X—H17G	0.9300
C6A—C5A	1.352 (2)	C17X—H17H	0.9300
C6A—H6AA	0.9300	C15Y—C16Y	1.429 (11)
C3C—H3CA	0.9300	C15Y—H15I	0.9700
O3C—C12C	1.4280 (16)	C15Y—H15J	0.9700
C3B—H3BA	0.9300	C16Y—C17Y	1.174 (12)
C13C—C12C	1.4972 (19)	C16Y—H16E	0.9300
C13C—C14C	1.514 (2)	C17Y—H17I	0.9300
C13C—H13A	0.9700	C17Y—H17J	0.9300
C13C—H13B	0.9700	C15Z—C16Z	1.545 (12)
C13B—C12B	1.501 (2)	C15Z—H15K	0.9700
C13B—C14B	1.516 (2)	C15Z—H15L	0.9700
C13B—H13C	0.9700	C16Z—C17Z	1.344 (10)
C13B—H13D	0.9700	C16Z—H16F	0.9300
C3A—H3AA	0.9300	C17Z—H17K	0.9300
C10B—H10A	0.9300	C17Z—H17L	0.9300
O3A—C12A	1.4183 (18)		
			
C10C—C11C—C2C	120.14 (12)	C8A—C9A—C10A	122.75 (12)
C10C—C11C—H11A	119.9	C8A—C9A—C4A	119.25 (12)
C2C—C11C—H11A	119.9	C10A—C9A—C4A	117.99 (12)
C1A—O2A—H1	118.0	O3C—C12C—C13C	108.49 (11)
C3B—C2B—C11B	119.20 (11)	O3C—C12C—H12A	110.0
C3B—C2B—C1B	120.41 (12)	C13C—C12C—H12A	110.0
C11B—C2B—C1B	120.40 (11)	O3C—C12C—H12B	110.0
C10B—C11B—C2B	120.37 (12)	C13C—C12C—H12B	110.0
C10B—C11B—H11B	119.8	H12A—C12C—H12B	108.4
C2B—C11B—H11B	119.8	O3A—C12A—C13A	107.21 (13)
C1B—O2B—H2	109.4	O3A—C12A—H12C	110.3
C8B—C9B—C10B	122.61 (12)	C13A—C12A—H12C	110.3
C8B—C9B—C4B	119.53 (12)	O3A—C12A—H12D	110.3
C10B—C9B—C4B	117.86 (12)	C13A—C12A—H12D	110.3
C3A—C2A—C11A	119.26 (12)	H12C—C12A—H12D	108.5
C3A—C2A—C1A	120.05 (12)	O3B—C12B—C13B	107.38 (13)
C11A—C2A—C1A	120.69 (11)	O3B—C12B—H12E	110.2
C8C—C9C—C10C	122.28 (12)	C13B—C12B—H12E	110.2
C8C—C9C—C4C	119.52 (12)	O3B—C12B—H12F	110.2
C10C—C9C—C4C	118.20 (11)	C13B—C12B—H12F	110.2
C1C—O2C—H3	118.4	H12E—C12B—H12F	108.5
C3B—C4B—C5B	122.20 (12)	C7A—C8A—C9A	119.82 (13)
C3B—C4B—C9B	119.36 (12)	C7A—C8A—H8AA	120.1
C5B—C4B—C9B	118.44 (12)	C9A—C8A—H8AA	120.1
O3C—C7C—C8C	125.36 (12)	C15Z—C14C—C13C	113.8 (6)
O3C—C7C—C6C	114.22 (11)	C15Z—C14C—C15C	2.8 (10)
C8C—C7C—C6C	120.42 (12)	C13C—C14C—C15C	112.4 (2)
C3C—C2C—C11C	119.53 (12)	C15Z—C14C—H14E	110.5
C3C—C2C—C1C	120.48 (12)	C13C—C14C—H14E	109.1
C11C—C2C—C1C	119.99 (11)	C15C—C14C—H14E	109.1
C15A—C14A—C13A	116.7 (3)	C15Z—C14C—H14F	106.3
C15A—C14A—C15X	14.5 (6)	C13C—C14C—H14F	109.1
C13A—C14A—C15X	102.3 (5)	C15C—C14C—H14F	109.1
C15A—C14A—H14A	108.1	H14E—C14C—H14F	107.9
C13A—C14A—H14A	108.1	C15Z—C14C—H14K	101.5
C15X—C14A—H14A	113.5	C13C—C14C—H14K	108.9
C15A—C14A—H14B	108.1	C15C—C14C—H14K	99.9
C13A—C14A—H14B	108.1	H14E—C14C—H14K	10.8
C15X—C14A—H14B	117.0	H14F—C14C—H14K	117.2
H14A—C14A—H14B	107.3	C15Z—C14C—H14L	115.5
C15A—C14A—H14G	101.7	C13C—C14C—H14L	108.9
C13A—C14A—H14G	111.5	C15C—C14C—H14L	118.3
C15X—C14A—H14G	110.6	H14E—C14C—H14L	97.9
H14A—C14A—H14G	110.6	H14F—C14C—H14L	11.2
H14B—C14A—H14G	6.5	H14K—C14C—H14L	107.7
C15A—C14A—H14H	105.1	C15B—C14B—C13B	115.8 (2)
C13A—C14A—H14H	111.8	C15B—C14B—C15Y	12.7 (5)
C15X—C14A—H14H	111.2	C13B—C14B—C15Y	103.1 (4)
H14A—C14A—H14H	3.8	C15B—C14B—H14C	108.3
H14B—C14A—H14H	106.5	C13B—C14B—H14C	108.3
H14G—C14A—H14H	109.4	C15Y—C14B—H14C	114.5
C3A—C4A—C5A	121.96 (12)	C15B—C14B—H14D	108.3
C3A—C4A—C9A	119.21 (12)	C13B—C14B—H14D	108.3
C5A—C4A—C9A	118.82 (12)	C15Y—C14B—H14D	114.8
C10A—C11A—C2A	120.41 (12)	H14C—C14B—H14D	107.4
C10A—C11A—H11C	119.8	C15B—C14B—H14I	104.0
C2A—C11A—H11C	119.8	C13B—C14B—H14I	111.3
O3B—C7B—C8B	126.05 (13)	C15Y—C14B—H14I	110.6
O3B—C7B—C6B	113.55 (12)	H14C—C14B—H14I	108.9
C8B—C7B—C6B	120.40 (13)	H14D—C14B—H14I	4.4
C3C—C4C—C5C	122.33 (12)	C15B—C14B—H14J	104.8
C3C—C4C—C9C	119.25 (11)	C13B—C14B—H14J	111.3
C5C—C4C—C9C	118.42 (12)	C15Y—C14B—H14J	111.3
O3A—C7A—C8A	126.28 (14)	H14C—C14B—H14J	3.7
O3A—C7A—C6A	112.93 (12)	H14D—C14B—H14J	107.9
C8A—C7A—C6A	120.79 (13)	H14I—C14B—H14J	109.2
C5B—C6B—C7B	120.57 (13)	C16A—C15A—C14A	115.8 (4)
C5B—C6B—H6BA	119.7	C16A—C15A—H15A	108.3
C7B—C6B—H6BA	119.7	C14A—C15A—H15A	108.3
C5C—C6C—C7C	120.46 (12)	C16A—C15A—H15B	108.3
C5C—C6C—H6CA	119.8	C14A—C15A—H15B	108.3
C7C—C6C—H6CA	119.8	H15A—C15A—H15B	107.4
C7B—O3B—C12B	118.92 (11)	C17A—C16A—C15A	123.2 (4)
C5A—C6A—C7A	120.29 (13)	C17A—C16A—H16A	118.4
C5A—C6A—H6AA	119.9	C15A—C16A—H16A	118.4
C7A—C6A—H6AA	119.9	C16A—C17A—H17A	120.0
C2C—C3C—C4C	121.35 (12)	C16A—C17A—H17B	120.0
C2C—C3C—H3CA	119.3	H17A—C17A—H17B	120.0
C4C—C3C—H3CA	119.3	C16B—C15B—C14B	118.4 (4)
C7C—O3C—C12C	117.80 (10)	C16B—C15B—H15C	107.7
C2B—C3B—C4B	121.58 (12)	C14B—C15B—H15C	107.7
C2B—C3B—H3BA	119.2	C16B—C15B—H15D	107.7
C4B—C3B—H3BA	119.2	C14B—C15B—H15D	107.7
C12C—C13C—C14C	111.61 (12)	H15C—C15B—H15D	107.1
C12C—C13C—H13A	109.3	C17B—C16B—C15B	125.8 (5)
C14C—C13C—H13A	109.3	C17B—C16B—H16B	117.1
C12C—C13C—H13B	109.3	C15B—C16B—H16B	117.1
C14C—C13C—H13B	109.3	C16B—C17B—H17C	120.0
H13A—C13C—H13B	108.0	C16B—C17B—H17D	120.0
C12B—C13B—C14B	112.58 (14)	H17C—C17B—H17D	120.0
C12B—C13B—H13C	109.1	C16C—C15C—C14C	113.2 (4)
C14B—C13B—H13C	109.1	C16C—C15C—H15E	108.9
C12B—C13B—H13D	109.1	C14C—C15C—H15E	108.9
C14B—C13B—H13D	109.1	C16C—C15C—H15F	108.9
H13C—C13B—H13D	107.8	C14C—C15C—H15F	108.9
C2A—C3A—C4A	121.75 (12)	H15E—C15C—H15F	107.8
C2A—C3A—H3AA	119.1	C17C—C16C—C15C	128.3 (5)
C4A—C3A—H3AA	119.1	C17C—C16C—H16C	115.9
C11B—C10B—C9B	121.60 (12)	C15C—C16C—H16C	115.9
C11B—C10B—H10A	119.2	C16C—C17C—H17E	120.0
C9B—C10B—H10A	119.2	C16C—C17C—H17F	120.0
C7A—O3A—C12A	118.97 (12)	H17E—C17C—H17F	120.0
C11C—C10C—C9C	121.49 (12)	C16X—C15X—C14A	110.2 (10)
C11C—C10C—H10B	119.3	C16X—C15X—H15G	109.6
C9C—C10C—H10B	119.3	C14A—C15X—H15G	109.6
C11A—C10A—C9A	121.37 (13)	C16X—C15X—H15H	109.6
C11A—C10A—H10C	119.3	C14A—C15X—H15H	109.6
C9A—C10A—H10C	119.3	H15G—C15X—H15H	108.1
C6B—C5B—C4B	120.96 (13)	C17X—C16X—C15X	143.1 (16)
C6B—C5B—H5BA	119.5	C17X—C16X—H16D	108.4
C4B—C5B—H5BA	119.5	C15X—C16X—H16D	108.4
C6C—C5C—C4C	120.94 (13)	C16X—C17X—H17G	120.0
C6C—C5C—H5CA	119.5	C16X—C17X—H17H	120.0
C4C—C5C—H5CA	119.5	H17G—C17X—H17H	120.0
C14A—C13A—C12A	112.81 (14)	C16Y—C15Y—C14B	105.1 (11)
C14A—C13A—H13E	109.0	C16Y—C15Y—H15I	110.7
C12A—C13A—H13E	109.0	C14B—C15Y—H15I	110.7
C14A—C13A—H13F	109.0	C16Y—C15Y—H15J	110.7
C12A—C13A—H13F	109.0	C14B—C15Y—H15J	110.7
H13E—C13A—H13F	107.8	H15I—C15Y—H15J	108.8
O1A—C1A—O2A	122.42 (12)	C17Y—C16Y—C15Y	142 (2)
O1A—C1A—C2A	119.90 (12)	C17Y—C16Y—H16E	108.8
O2A—C1A—C2A	117.68 (11)	C15Y—C16Y—H16E	108.8
O1C—C1C—O2C	122.79 (12)	C16Y—C17Y—H17I	120.0
O1C—C1C—C2C	119.71 (12)	C16Y—C17Y—H17J	120.0
O2C—C1C—C2C	117.50 (11)	H17I—C17Y—H17J	120.0
C7B—C8B—C9B	120.08 (13)	C14C—C15Z—C16Z	114.7 (11)
C7B—C8B—H8BA	120.0	C14C—C15Z—H15K	108.6
C9B—C8B—H8BA	120.0	C16Z—C15Z—H15K	108.6
C6A—C5A—C4A	121.02 (13)	C14C—C15Z—H15L	108.6
C6A—C5A—H5AA	119.5	C16Z—C15Z—H15L	108.6
C4A—C5A—H5AA	119.5	H15K—C15Z—H15L	107.6
O1B—C1B—O2B	122.96 (11)	C17Z—C16Z—C15Z	111.5 (10)
O1B—C1B—C2B	119.68 (12)	C17Z—C16Z—H16F	124.3
O2B—C1B—C2B	117.36 (11)	C15Z—C16Z—H16F	124.3
C7C—C8C—C9C	120.19 (12)	C16Z—C17Z—H17K	120.0
C7C—C8C—H8CA	119.9	C16Z—C17Z—H17L	120.0
C9C—C8C—H8CA	119.9	H17K—C17Z—H17L	120.0
			
C3B—C2B—C11B—C10B	1.7 (2)	C3C—C2C—C1C—O1C	−179.67 (11)
C1B—C2B—C11B—C10B	−178.59 (11)	C11C—C2C—C1C—O1C	0.63 (19)
C8B—C9B—C4B—C3B	−179.12 (10)	C3C—C2C—C1C—O2C	−0.28 (19)
C10B—C9B—C4B—C3B	1.80 (19)	C11C—C2C—C1C—O2C	−179.98 (11)
C8B—C9B—C4B—C5B	1.64 (19)	O3B—C7B—C8B—C9B	179.10 (12)
C10B—C9B—C4B—C5B	−177.44 (11)	C6B—C7B—C8B—C9B	−1.0 (2)
C10C—C11C—C2C—C3C	1.3 (2)	C10B—C9B—C8B—C7B	178.42 (12)
C10C—C11C—C2C—C1C	−179.04 (12)	C4B—C9B—C8B—C7B	−0.6 (2)
C3A—C2A—C11A—C10A	0.7 (2)	C7A—C6A—C5A—C4A	−0.4 (2)
C1A—C2A—C11A—C10A	−179.50 (12)	C3A—C4A—C5A—C6A	−179.89 (12)
C8C—C9C—C4C—C3C	−179.12 (11)	C9A—C4A—C5A—C6A	−0.5 (2)
C10C—C9C—C4C—C3C	1.57 (18)	C3B—C2B—C1B—O1B	−179.09 (11)
C8C—C9C—C4C—C5C	1.91 (19)	C11B—C2B—C1B—O1B	1.21 (19)
C10C—C9C—C4C—C5C	−177.40 (11)	C3B—C2B—C1B—O2B	0.80 (19)
O3B—C7B—C6B—C5B	−178.51 (12)	C11B—C2B—C1B—O2B	−178.90 (11)
C8B—C7B—C6B—C5B	1.5 (2)	O3C—C7C—C8C—C9C	178.08 (11)
O3C—C7C—C6C—C5C	−177.60 (12)	C6C—C7C—C8C—C9C	−2.0 (2)
C8C—C7C—C6C—C5C	2.5 (2)	C10C—C9C—C8C—C7C	179.09 (12)
C8B—C7B—O3B—C12B	0.9 (2)	C4C—C9C—C8C—C7C	−0.2 (2)
C6B—C7B—O3B—C12B	−179.03 (12)	C11A—C10A—C9A—C8A	−179.90 (12)
O3A—C7A—C6A—C5A	−179.09 (12)	C11A—C10A—C9A—C4A	−0.7 (2)
C8A—C7A—C6A—C5A	0.9 (2)	C3A—C4A—C9A—C8A	−179.73 (11)
C11C—C2C—C3C—C4C	−0.40 (19)	C5A—C4A—C9A—C8A	0.88 (19)
C1C—C2C—C3C—C4C	179.90 (10)	C3A—C4A—C9A—C10A	1.07 (18)
C5C—C4C—C3C—C2C	177.91 (12)	C5A—C4A—C9A—C10A	−178.32 (11)
C9C—C4C—C3C—C2C	−1.01 (19)	C7C—O3C—C12C—C13C	−175.46 (11)
C8C—C7C—O3C—C12C	−2.2 (2)	C14C—C13C—C12C—O3C	−175.34 (12)
C6C—C7C—O3C—C12C	177.91 (12)	C7A—O3A—C12A—C13A	−173.54 (12)
C11B—C2B—C3B—C4B	−0.8 (2)	C14A—C13A—C12A—O3A	−177.02 (13)
C1B—C2B—C3B—C4B	179.53 (10)	C7B—O3B—C12B—C13B	−177.49 (12)
C5B—C4B—C3B—C2B	178.22 (11)	C14B—C13B—C12B—O3B	−175.95 (12)
C9B—C4B—C3B—C2B	−1.00 (19)	O3A—C7A—C8A—C9A	179.48 (12)
C11A—C2A—C3A—C4A	−0.34 (19)	C6A—C7A—C8A—C9A	−0.6 (2)
C1A—C2A—C3A—C4A	179.86 (11)	C10A—C9A—C8A—C7A	178.81 (12)
C5A—C4A—C3A—C2A	178.82 (11)	C4A—C9A—C8A—C7A	−0.3 (2)
C9A—C4A—C3A—C2A	−0.55 (19)	C12C—C13C—C14C—C15Z	179.2 (9)
C2B—C11B—C10B—C9B	−0.9 (2)	C12C—C13C—C14C—C15C	176.5 (3)
C8B—C9B—C10B—C11B	−179.95 (12)	C12B—C13B—C14B—C15B	178.4 (3)
C4B—C9B—C10B—C11B	−0.9 (2)	C12B—C13B—C14B—C15Y	178.6 (5)
C8A—C7A—O3A—C12A	−3.8 (2)	C13A—C14A—C15A—C16A	−172.6 (3)
C6A—C7A—O3A—C12A	176.22 (12)	C15X—C14A—C15A—C16A	180 (3)
C2C—C11C—C10C—C9C	−0.7 (2)	C14A—C15A—C16A—C17A	−125.5 (5)
C8C—C9C—C10C—C11C	179.97 (12)	C13B—C14B—C15B—C16B	−171.1 (3)
C4C—C9C—C10C—C11C	−0.7 (2)	C15Y—C14B—C15B—C16B	−172 (4)
C2A—C11A—C10A—C9A	−0.2 (2)	C14B—C15B—C16B—C17B	−123.6 (5)
C7B—C6B—C5B—C4B	−0.5 (2)	C15Z—C14C—C15C—C16C	77 (17)
C3B—C4B—C5B—C6B	179.68 (12)	C13C—C14C—C15C—C16C	−163.7 (4)
C9B—C4B—C5B—C6B	−1.1 (2)	C14C—C15C—C16C—C17C	−126.8 (5)
C7C—C6C—C5C—C4C	−0.7 (2)	C15A—C14A—C15X—C16X	−12 (2)
C3C—C4C—C5C—C6C	179.59 (12)	C13A—C14A—C15X—C16X	175.0 (9)
C9C—C4C—C5C—C6C	−1.5 (2)	C14A—C15X—C16X—C17X	−33 (3)
C15A—C14A—C13A—C12A	179.8 (2)	C15B—C14B—C15Y—C16Y	2 (3)
C15X—C14A—C13A—C12A	−178.2 (6)	C13B—C14B—C15Y—C16Y	−177.2 (10)
C3A—C2A—C1A—O1A	179.96 (12)	C14B—C15Y—C16Y—C17Y	−83 (4)
C11A—C2A—C1A—O1A	0.2 (2)	C13C—C14C—C15Z—C16Z	164.4 (9)
C3A—C2A—C1A—O2A	−0.32 (19)	C15C—C14C—C15Z—C16Z	−134 (18)
C11A—C2A—C1A—O2A	179.88 (11)	C14C—C15Z—C16Z—C17Z	110.9 (13)

### Hydrogen-bond geometry (Å, º)

**Table:**

*D*—H···*A*	*D*—H	H···*A*	*D*···*A*	*D*—H···*A*
O2*A*—H1···O1*C*^i^	0.97	1.65	2.6150 (16)	174
O2*B*—H2···O1*B*^ii^	0.85	1.80	2.6342 (15)	168
O2*C*—H3···O1*A*^i^	0.93	1.69	2.6133 (16)	177
C6*A*—H6*AA*···O1*A*^iii^	0.93	2.50	3.3032 (19)	144
C6*B*—H6*BA*···O1*B*^iii^	0.93	2.56	3.3666 (19)	145
C6*C*—H6*CA*···O1*C*^iii^	0.93	2.56	3.3547 (18)	144
C5*A*—H5*AA*···O2*C*^iv^	0.93	2.59	3.421 (2)	149
C5*B*—H5*BA*···O2*B*^v^	0.93	2.65	3.520 (2)	156
C5*C*—H5*CA*···O2*A*^iv^	0.93	2.61	3.472 (2)	154
C17*B*—H17*C*···*Cg*1^vi^	0.93	2.93	3.736 (5)	146

Symmetry codes: (i) −*x*+2, −*y*+2, −*z*+1; (ii) −*x*+3, −*y*+2, −*z*; (iii) *x*−1, *y*, *z*; (iv) −*x*+1, −*y*+2, −*z*+1; (v) −*x*+2, −*y*+2, −*z*; (vi) −*x*+1, −*y*+1, −*z*+1.
